# Implementing a fax referral program for quitline smoking cessation services in urban health centers: a qualitative study

**DOI:** 10.1186/1471-2296-10-81

**Published:** 2009-12-17

**Authors:** Jennifer Cantrell, Donna Shelley

**Affiliations:** 1 Department of Cariology and Comprehensive Care, School of Medicine and Dentistry, New York University, New York, NY, USA; 2 Mailman School of Public Health, Columbia University, New York, NY, USA and Public Health Solutions, National Development Research Institutes, Inc., Behavior Science Training Program; 3 Department of Cariology and Comprehensive Care and Department of General Internal Medicine, School of Medicine and Dentistry, New York University, New York, NY USA

## Abstract

**Background:**

Fax referral services that connect smokers to state quitlines have been implemented in 49 U.S. states and territories and promoted as a simple solution to improving smoker assistance in medical practice. This study is an in-depth examination of the systems-level changes needed to implement and sustain a fax referral program in primary care.

**Methods:**

The study involved implementation of a fax referral system paired with a chart stamp prompting providers to identify smoking patients, provide advice to quit and refer interested smokers to a state-based fax quitline. Three focus groups (n = 26) and eight key informant interviews were conducted with staff and physicians at two clinics after the intervention. We used the Chronic Care Model as a framework to analyze the data, examining how well the systems changes were implemented and the impact of these changes on care processes, and to develop recommendations for improvement.

**Results:**

Physicians and staff described numerous benefits of the fax referral program for providers and patients but pointed out significant barriers to full implementation, including the time-consuming process of referring patients to the Quitline, substantial patient resistance, and limitations in information and care delivery systems for referring and tracking smokers. Respondents identified several strategies for improving integration, including simplification of the referral form, enhanced teamwork, formal assignment of responsibility for referrals, ongoing staff training and patient education. Improvements in Quitline feedback were needed to compensate for clinics' limited internal information systems for tracking smokers.

**Conclusions:**

Establishing sustainable linkages to quitline services in clinical sites requires knowledge of existing patterns of care and tailored organizational changes to ensure new systems are prioritized, easily integrated into current office routines, formally assigned to specific staff members, and supported by internal systems that ensure adequate tracking and follow up of smokers. Ongoing staff training and patient self-management techniques are also needed to ease the introduction of new programs and increase their acceptability to smokers.

## Background

Each visit a smoker makes to their primary care provider represents an opportunity to offer treatment for tobacco dependence. Effective treatment, as defined by the Public Health Service Guideline, includes asking about tobacco use, advising smokers to quit, assessing readiness, providing cessation assistance and arranging for follow up (5As) [1]. National surveys indicate that clinicians are increasingly screening for tobacco use and offering brief advice; however, rates of assistance are still too low [2]. One recommended strategy to improve smoker assistance in primary care settings is to link practices to external counseling resources, such as statewide telephone quitlines [3-7].

Telephone quitlines have proven effective in increasing smoking cessation [1] and are available in all 50 states of the U.S. Quitlines in 49 U.S. states and territories provide services that allow health care providers to refer patients for proactive telephone counseling through a fax referral system [8]; in New York State (NYS), this is called the Fax-to-Quit program. Recent studies suggest that fax referrals may provide a feasible and low-cost mechanism for enrolling tobacco users in statewide telephone counseling [9-16]. Given the wide-scale implementation of fax referral services nationally, an understanding of system changes required to implement and sustain these programs in health care service settings is critical.

As with other systems improvements, a fax referral system necessitates a series of changes in care processes. These include the establishment of simple and efficient clinical pathways for consistently identifying, advising and assessing smokers, ensuring that referrals from the clinic are sent to and received at the quitline in a timely manner and, likewise, that information from the quitline on smokers' progress is received and incorporated into clinic systems for appropriate follow up [2]. The sustainability of these new systems and practice procedures requires that they be integrated into patient and clinic workflow in a seamless manner that does not overly burden staff, clinic, or patient resources.

This paper presents the results of the qualitative component of a mixed-methods study that evaluated the impact of a Fax-to-Quit referral system plus an expanded "vital sign" chart stamp compared to a chart stamp alone on provider adherence to the recommended 5As and referrals to the Fax-to-Quit service in primary care clinics. This is the first in-depth examination of the systems-level care process changes needed to implement and sustain a fax referral program to state quitline services in the primary care practice setting. The purpose of this analysis was to examine how well the systems changes were implemented, the impact of these changes on care processes, and to develop recommendations to ensure the sustainability of effective systems.

## Methods

### Setting

**Figure 1:**
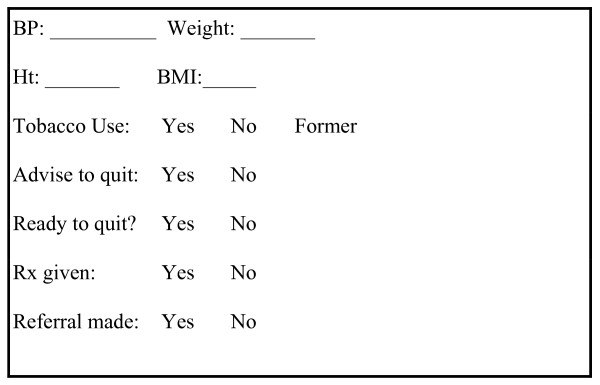
Chart stamp: Ask, Advise, Assess, Refer, Prescribe

The four clinics enrolled in this study (two intervention and two comparison clinics) were part of a system of outpatient clinics linked to New York Presbyterian Hospital Ambulatory Care Research Network (ACRN), a practice-based research network of 12 community health clinics. These clinics serve a primarily Hispanic low-income population. Prior to the study, as part of a quality improvement initiative to disseminate tobacco use treatment guidelines, the research team worked with all clinics within the ACRN to implement an expanded "vital sign" chart stamp, that prompted providers to ask patients about tobacco use, advise them to quit, assess readiness to quit, offer assistance and arrange follow-up. We divided the prompt to provide assistance and arrange follow-up into two components on the chart stamp: prescription given and referral made (see Figure 1). All of the sites were using paper-based charts at that time; therefore, the chart stamp was pre-printed on all patient encounter forms. The clinics had integrated the stamp into their services at least six months prior to this study.

**Figure 2:**
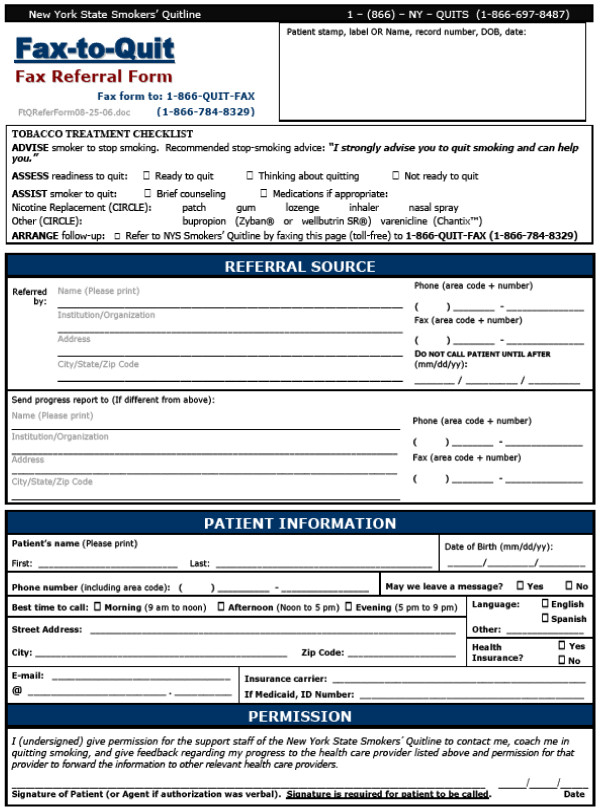
New York State Quitline (NYS) fax referral form

In intervention sites, the chart stamp was paired with the New York State Quitline (NYS) fax referral system (see Figure 2). Providers used the referral form to refer current smokers to the Fax-to-Quit program by faxing the form from the clinic to the state Quitline. Upon receipt of the faxed referral form, a trained Quitline counselor contacts the patient to offer customized cessation counseling, pharmacotherapy for eligible smokers and additional assistance. The Quitline then faxes a progress report on the patient's status back to the clinic for provider follow-up. The intervention was delivered over a four-month period in the two intervention sites and involved staff training on the Fax-to-Quit program as well as two site visits. In addition, providers received two emailed performance feedback reports.

### Data collection

We conducted key informant interviews with eight out of 11 primary care physicians. Of the three not participating, one had left the clinic, one was unable to schedule an interview within the period of the study, and one refused. The Principal Investigator or a doctoral-level research associate conducted the interviews. Each interview lasted approximately 45 minutes and was taped.

We also conducted three focus groups across the two sites with a total of 26 participants. Among participants, 54% were medical assistants (MAs), 27% were registered nurses, 15% worked as registration coordinators, patient financial advisors, or administrative assistants, and 4% were clinical social workers. A co-investigator facilitated the focus groups with assistance from a research assistant. The focus groups lasted approximately 60 minutes and were taped.

We developed semi-structured interview and focus group guides to direct the interviews, although participants were free to raise other issues they believed important. Topics addressed included how the chart stamp and Fax-to-Quit systems were used; the influence of the systems on tobacco use discussions, screening, and treatment; patients' responses; benefits and barriers to system use; and recommendations to streamline the systems.

Audiotapes of the focus groups were transcribed and entered into Atlas.ti qualitative data analysis software http://www.atlasti.com/. The study protocol was reviewed and approved by the Institutional Review Boards of Columbia University and New York University.

### Data analysis

We utilized the Chronic Care Model framework to identify and evaluate systems-level changes needed to implement the fax referral program. The Model is a framework for systems-focused quality improvement programs. While originally utilized to improve health delivery systems for chronic care management [17-21], it has more recently been used as a framework for addressing preventive care and risk behavior [22,23], including tobacco use [24,25]. The model includes four main components for health systems, as shown in Table 1: decision support, delivery system design, clinical information systems, and patient self-management. We reviewed the staff and physician interview data together and related the themes identified to these four components of the model to deductively code the interview and focus group transcripts, using the template organizing style [26]. We also examined key similarities and differences in the data by physician or staff status. We ended our coding by searching for exceptions that might disconfirm our analytic observations and included these in the results, and, lastly, re-reviewed the transcripts to verify the final analysis.

**Table 1:** Components of the Chronic Care Model for health systems

Definition and examples of CCM components
**Decision support**
Embedding evidence-based guidelines into daily clinical practice (e.g., systems prompts and reminders; clinical pocket guides)

**Delivery system design**
Defining staff roles and distributing tasks appropriately among health team members to deliver coordinated care (e.g. use of non-physician staff to deliver counseling and coordinate care; clearly defined provider roles and responsibilities)

**Clinical information systems**
Utilizing patient and population data to facilitate efficient care, tracking and follow up (e.g., electronic medical records; patient registries; patient status summaries)

**Patient self-management**
Empowering patients to manage their own health through education and self-management support strategies (e.g., tailored educational resources; psychosocial support)

## Results

### Decision support (see Table 2)

**Table 2:** Themes related to decision support and delivery system design

CCM component	Sample Quotes
**Decision support**	
Chart stamp as an efficient prompt to ask, advise and refer	"Asking on a regular follow up wouldn't really happen if the prompt wasn't there." (Physician)
	"It has made me more thorough. I feel like I have much more of a process to remind me." (Physician)
Fax-to-Quit referral form was lengthy and complicated	"That piece of paper [the fax referral form] is still more complicated than my patients can truly understand. You know, third grade level, at the most." (Physician)
	"The form should be made simpler because we do have a lot of patients to take care of, and some steps should be eliminated." (Medical Assistant)
Limitations with faxing	"You have to fax it, that's another step. Sometimes the fax machines here don't work. So you have to remember to go back and fax it." (Medical Assistant)

**Delivery system design**	
Chart stamp integrated into paper flow, MA/physician workflow and responsibilities	"I think the stamp is sustainable because we integrated it very well into the paper flow of the clinic and we also integrated it into the workflow." (Physician)
Chart stamp not integrated into nurse and social worker workflow or responsibilities	"I think for nursing, we don't ask this of the patients. That's why we don't use this. But if we see our patients that smoke, we try to advise the patient even though we are not using the chart stamp." (Nurse)
Fax-to-Quit form not integrated into paper flow or team responsibilities	"It [the chart stamp] was a system change that was integrated. Fax-to-Quit has not become a system change that's integrated. It's become another activity that the physician really still had to take on." (Physician)
	"That will probably be the next step if we can actually propel our Medical Assistants and say 'Okay, you can now have a conversation around that too."' (Physician)
Concerns about overburdening staff and staff resistance	"My Medical Assistants should have some time to do the Fax-to-Quit. I know they're busy. I don't ever think they're not doing something. But the front desk is even busier sometimes." (Physician)
Need for further Fax-to-Quit training	"They may have questions and we can't, you know, go through all the questions and really tell them what's going on, because, first of all, most of us are not thoroughly trained ourselves." (Medical Assistant)

MAs and physicians reported consistent use of the chart stamp for screening and counseling, and as a reminder to refer and prescribe. MAs documented the "ask" portion of the stamp at all visits with new and continuing patients, which occurred before patients saw the physician or a nurse. Physicians reported using the stamp primarily as a support tool to remind them of the patient's smoking status and to prompt further assessment and services for smokers. Of note, providers agreed that the chart stamp did not interrupt workflow or add time to the visit.

The Fax-to Quit referral was also viewed as a valuable resource for busy clinicians. In contrast to the chart stamp, however, the Fax-to-Quit referral form was not as easily integrated into the patient encounter. A barrier to using this system included the unanticipated lengthy process involved in explaining the program, which could take up to 15 minutes. Staff and physicians described the form as too complicated, particularly for the literacy level of the patient population, and they reported language barriers in explaining the Fax-to-Quit program to patients who did not speak English as their first language.

The emphasis on faxing also presented technological challenges, as clinics did not always have a reliable fax machine and the faxing process added staff time to the patient encounter. In addition to simplifying the form, staff suggested streamlining the faxing process within the clinic to save time and improve integration.

### Delivery system design (Table 2)

The goal of creating a team approach, in which all staff were knowledgeable about tobacco cessation policies and procedures and responsibility for tobacco treatment would be spread across multiple staff, was not completely realized. According to providers and staff, the chart stamp was better integrated into staff workflow than the Fax-to-Quit program because it was embedded in the encounter form and, therefore, easy to access. There was also clear role definition related to this system change: MAs were tasked to screen all patients using the chart stamp prompt and physicians to follow-up on the remaining items on the chart stamp.

The interviews did uncover an unanticipated gap in using the chart stamp to reach patients who may have bypassed the MA prior to seeing the physician or who were at the clinic to see a social worker. Nurses and social workers reported that forms they used with patients, such as for triage or patient education, did not include the stamp. As a result, patients who came in for a nurse or social worker visit and did not see a MA or doctor were not consistently screened, counseled, and referred.

In contrast to a team approach between the MA and physician in completing the chart stamp, the physician had primary responsibility for the Fax-to-Quit referrals. MAs and nurses were trained to make the Quitline referrals, but they did not consistently perform this task. Physicians explained the program to patients, filled out the form and often faxed it. At times they also had to leave the room to locate forms as they were not in the chart and not always in the exam rooms. Physicians felt this limited the long-term sustainability of the program.

The majority of physicians expressed a desire for more teamwork in providing tobacco treatment and referring smokers; however, they were reluctant to ask staff to do more due to concerns about overburdening staff and staff resistance. Physicians also believed that smoking cessation assistance fell well within the responsibilities of nursing staff as they were already providing education on other health issues.

One of the barriers to staff engagement was the perception that more training was needed to increase staff confidence and expertise in delivering tobacco use treatment. Once the topics of tobacco use and the availability of telephone counseling were raised, patients often asked questions that the MAs and nurses did not feel they could answer. Both staff and physicians felt that they could use more training on the motivational aspects of helping smokers quit and in answering questions about the Quitline. Physicians felt that additional training for MAs and nurses could increase referrals as well.

### Clinical information systems (see Table 3)

**Table 3:** Themes related to clinical information systems and patient self-management

Clinical information systems	
Need for internal tracking systems and staff to follow up with smokers	"How do we follow up - let's say the patient came in and he did this [the fax referral] with me. How do I know that they actually called you? That they actually did something? How am I going to remember the patient?" (Medical Assistant)
	"It would make sense to have a nurse in charge of picking up progress reports [from the Quitline] when they come in, and following up with patients. We just don't have the staff resources." (Physician)
Limited understanding of specifics of the Fax-to-Quit program	"There's a sheet that comes back, usually informing us they [patients] have not been contacted but in terms of knowing how things are actually working, I'm not so sure about that." (Nurse)
Need for improved external information from Quitline	"I'm all about seeing the big picture so if they [the Quitline] could give you a printout of all the people referred, it can give us a sense of scale. That would help with sustainability." (Physician)
	"If I got a summary sheet of the patients, and these were unable to contact, and how many we actually did contact, and if there was any set plan, or patients were sent patches. It'd be nice to see a summary of the 25 people that you referred." (Physician)
	"Beyond the quantitative, it might be nice to hear some qualitative feedback. Perhaps a case scenario where a real connection happened - an example of an optimal Fax-to-Quit patient interaction." (Physician)
**Patient self-management**	
Fax-to-Quit as a valuable resource for patients to manage their smoking	"I think there's value in it. I love the fact that someone did a follow up. A behavior modification with reinforcement might make a more effective process." (Physician)
Patients were skeptical of the Fax-to-Quit program	"I've asked them, 'Do you want to fill this document out?' But all the questions come up, and that's where it starts. They ask you over and over so you have to give them some time." (Medical Assistant)
	"There are definitely a lot of resistant people at the idea of somebody calling them. They're like 'No, no, no."' (Physician)
Need for patient education	"You would get a lot more response if you had one person in the front [waiting room], you might get this [Fax-to-Quit form] filled out. It's a lot simpler than us having to rush through it." (Medical Assistant)
	"The waiting period for the patients after they see us, the MAs, to see the doctor, it could be lengthy. And it could be used to educate the patient." (Medical Assistant)

The lack of internal information systems that tracked smokers in the practice longitudinally and external information systems that provided useful feedback from the Quitline to clinicians on the progress of referred smokers was a barrier to consistent guideline implementation. The sites did not have an internal tracking system for generating aggregated data on tobacco use treatments provided to smoking patients or for quickly retrieving and charting progress reports that were faxed back from the Quitline; as a result, clinicians were not able to easily track and conduct timely follow-up with smokers who received a fax referral. Participants also pointed out that staff constraints made it difficult to assign staff to follow up with smokers between clinic visits.

Respondents also felt that the Quitline progress reports were of minimal use. Participants expressed limited understanding about the specifics of the Fax-to-Quit program and frustration at the seemingly low rates of Quitline contact of patients. Physicians and staff suggested several ways to improve the usefulness of Quitline progress reports to better assist them with patient management. For example, physicians wanted summary reports that described how the Quitline counselors intervened with their patients, including suggestions for follow-up care.

### Patient self-management (Table 3)

Physicians indicated that the Fax-to-Quit program was an important patient resource to encourage self-management. They believed it served as an important educational resource, a behavior change program to help with quitting, and a message to patients that smoking cessation was a priority.

Although there were some positive responses from patients who had received the calls and medication from the Quitline, providers reported significant patient resistance to receiving a call at home from a stranger and skepticism about providing the information required on the referral form. Overcoming this resistance added significant time to completing the referral process.

Respondents believed that greater patient knowledge about the Fax-to-Quit and the benefits of the program would lead to higher receptivity when asked to participate. They suggested taking advantage of patient flow and the long waiting times for patients to see their doctor to educate patients on the Fax-to-Quit program, which could reduce time spent explaining the program during the visit. Staff also described a previous program that enrolled patients in Medicaid managed care programs in the waiting room and indicated that a similar approach might work for the Fax-to-Quit program.

## Discussion

As fax referral programs linking state quitlines to local health care providers are implemented in the U.S. nationwide, ask-advise-refer models supported by these programs have been promoted as a solution to enhance and simplify adoption of the 5As for improving tobacco treatment in primary care [3-7]. Findings from our study demonstrate that even such seemingly straightforward solutions for improving tobacco treatment must overcome significant organizational barriers in primary care practice to ensure full utilization and sustainability. Physicians and staff described numerous benefits of the chart stamp and Fax-to-Quit systems for providers and patients alike, but pointed out gaps in implementation that needed to be addressed to guarantee full integration into clinic and patient flow. The time-consuming process of referring patients to the Quitline combined with substantial patient resistance and limitations in information and care delivery systems raised questions about the long-term sustainability of the fax referral program in these clinics. Yet respondents identified several strategies for improving integration through tool simplification, enhanced teamwork and training, improved Quitline feedback, and patient education.

### Decision support

Adding new functions that are not well integrated into office routines can place a significant burden on overcommitted primary care practices. The relatively seamless integration of the chart stamp versus the fax referral form illustrated this difference. The chart stamp was included as part of the vital signs in the encounter form that was already used by MAs and physicians, and thus it did not require an additional step in the care process. While adding the stamp to the nurse and social worker forms was still needed to ensure full integration, respondents viewed the new chart stamp system as a helpful prompt that did not extend the visit time. In contrast, the fax referral form was cumbersome and the faxing process added a time-consuming new procedure to office routines. Staff suggested further simplification of the form to minimize respondent burden and modifications for patients with lower literacy levels to help in explaining the program to patients.

Web-based fax referral is an additional option that, if implemented efficiently, may increase referral rates. Sherman et al. found that transitioning from a multi-question consult referral that took one-minute to a simple web-based "two-click" referral, taking only seconds, in Veterans Health Administration clinics generated an overwhelming increase in smoker referrals for the California Smokers' Helpline [27]. The NYS Fax-to-Quit program currently offers a web-based option for providers using an online template that is similar to the hard copy referral, but less than 2% of provider referrals currently come from this source (P. Bax, personal communication, June 18, 2009). As part of a larger academic institution, these community health clinics faced unique barriers to using the web to transfer patient information, including an electronic hospital firewall that prevented clinics from sending patient data to outside sources and privacy concerns related to the Health Insurance Portability and Accountability Act provisions.

### Delivery system design

Variation in delivery of the fax referral among non-physician staff reflected lack of a consistent policy with clearly designated roles and formal accountability for tobacco referrals. Integrating a team-oriented approach for preventive care and treatment, particularly in resource-constrained settings, often requires substantive delivery system redesign as well as prioritizing among personnel at all levels [28-30]. The fax intervention may have been facilitated by enlisting visible internal support from top leadership at the ACRN to promote and assign responsibilities to specific staff for performing fax referrals. The chart system implementation was part of a broader high priority quality improvement initiative conducted across the entire ACRN and included formally redefining the MA role to conduct tobacco use screening. In contrast, the fax referral intervention engaged clinic-level leadership at the two study clinics to encourage staff to refer patients and training was provided, yet the Fax-to-Quit intervention was still viewed by staff and clinic leadership as another outside initiative among many, and official responsibilities for referral and faxing were not assigned. The lack of formal responsibility, limited priority, and slim staffing margins undermined efforts to spread referral tasks across non-physician staff. As a result, the responsibility for the Fax-to-Quit fell almost entirely on the physician. Similar to other studies [31,32], doctors expressed challenges offering the time-consuming referral to every eligible smoker during brief clinical encounters.

In developing a teamwork approach, further efforts were needed to engage non-physician clinic staff at all levels through additional education on tobacco and the Fax-to-Quit program. While staff received a total of 90 minutes of training as part of the quality improvement initiative and the Fax-to-Quit intervention, employee turnover, patients' Quitline questions, and patients' personal issues that often arose during tobacco discussions highlighted the need for relatively comprehensive education with regular booster sessions [33,34]. Such training should include basic motivational interviewing techniques, as well as instructions on brief counseling and referral techniques and explicit scripting of Quitline services to support the goal of the referral model, which is designed to cut down on the amount of time clinicians counsel patients by re-directing smokers to outside specialists [4,35]. Trainings should also include front-line staff, who can be incorporated into the tobacco treatment team for such critical tasks as faxing, coordinating follow up, or re-filing progress reports. Research suggests that ongoing behavioral counseling training and improved knowledge of community resources among clinic staff may be particularly important for sustaining referral linkages between clinics and external organizations [33].

### Clinical information systems

The lack of internal clinical information systems to provide timely information on referred smokers was a severe limitation to the sustainability of the referral program. Although Quitline progress reports provided individual-level information on referred smokers' status, the sites had no internal tracking system and limited resources to follow up with smokers the Quitline was unable to contact or who had set a quit date and received medications. The clinics needed in-house real-time information on the tobacco treatment status of smoking patients to adequately track referred patients. Since the time of this study, some ACRN clinics have transitioned to an electronic medical record (EMR) system. While EMRs can help practices track and monitor the delivery of tobacco services to patients [25], similar issues must still be addressed, including the development of efficient templates in the electronic record for documenting tobacco screening and intervention, policies delegating responsibility for data entry and follow-up, and integration into current clinical routines [24,36-38]. Sites without EMRs can implement patient registries as a potential option for managing smoking patients [19]. A simple paper-based registry to incorporate data from separate sources (e.g., patient charts, fax referral forms, Quitline progress reports, prescriptions) can be effective if designed with minimal response burden in mind and a staff member is identified for updating and maintenance [34].

Tighter integration between the clinic and the Fax-to-Quit program was needed to make use of the external information provided by the Quitline to clinics on their smoking patients. Most staff and physicians reported uncertainty on the specifics of the Fax-to-Quit program. While more intensive and continuous training may partially remedy this problem, further publicity efforts and outreach may be needed. The Quitline has worked with NYS-funded cessation centers, and increasingly, with provider organizations and health care plans to publicize the Fax-to-Quit program. In addition to these general efforts, targeted outreach to specific clinic networks and clinic staff at all levels could improve knowledge of this valuable program. Information from the Quitline could also be improved through the use of summary reports to individual clinicians on the progress of all smokers referred, which can reduce clinics' paperwork and faxing burden and would be particularly helpful for sites without internal systems to aggregate information on smoking patients.

Providers also reported seemingly low rates of Quitline contact of patients. Studies of fax referral programs in health care settings report quitline rates of reaching patients ranging from approximately 40-70% [9-16]. The reach rate in this study for patients referred from the two clinics during the intervention period was approximately 41% (NYS DOH, unpublished data, 2007), on the low end compared to these studies and slightly lower than the average NYS Quitline reach rate of 53% for fax referrals (NYS DOH, unpublished data, 2009). Our rates were similar to Mahabee-Gittens et al., who reported reach rates of 42% among a transient patient population fax referred through emergency departments [14]. Further research and collaboration with providers to develop new strategies for contacting highly mobile populations may be needed to improve patient connections. To improve contact rates, experts also recommend clinical targeting of only those smokers who are ready to quit [16]. Lastly, Quitlines must be adequately resources and sustainable, [39] utilizing funding from multiple sources if necessary, [40] to manage increases in demand following the introduction of fax referral systems and to implement outreach campaigns to providers, as discussed above.

### Patient self-management

According to providers, patient resistance and unfamiliarity with the Fax-to-Quit program was a significant barrier to referral in this study. This was in sharp contrast to recent reports from the national Prescription for Health study, which found that patients responded enthusiastically to new health behavior change resources [29]. Our findings point to the need for further outreach in medical and other settings tailored to diverse patient populations. Direct-to-consumer marketing on the Fax-to-Quit program, available either in the clinic waiting room or through other outside venues, can enhance the appeal of and demand for quitline services, particularly among disadvantaged groups [41,42]; such marketing will also benefit clinicians by reducing the amount of time needed to explain the program to patients. Further, increased consumer demand for quitline programs may have a synergistic effect: research indicates that providers are more likely to offer referrals if they believe the referral source is perceived as valuable by patients [33].

Data from this study come from clinicians and staff members of two health clinics serving a predominantly low-income, ethnic minority community in a large urban city in the U.S. To the extent that respondents and practices differ from the larger population of primary care practices and their employees in the U.S. and other countries in approaches to tobacco use management, our findings may have limited generalizability. The care components of the CCM model, however, can be applied across clinical settings. While specific care process changes needed for effective fax referral systems may depend somewhat on site leadership as well as system and population characteristics, consistent themes such as time, staff and resource needs for building and sustaining tobacco referral infrastructures [31,33,38] are likely to be relevant across primary care practices in developed countries' health care settings. Further, recommendations for improvements to quitline interactions with health care providers may apply to fax referral programs in other states or locales. Lastly, the nature of interviews and focus groups provides information only on aspects of clinical systems of which participants were aware; clinic observations or patient interviews might reveal additional characteristics of clinic systems and patient interactions that may impact systems implementation.

## Conclusions

Nationwide implementation of fax referral systems linking patients in clinics to local proactive quitlines offers a concrete strategy for assisting smokers. However, our findings illustrate the challenges of integrating and maintaining such systems. Establishing sustainable linkages to quitline services in clinical sites requires knowledge of existing patterns of care and tailored organizational changes to ensure new systems are prioritized, easily integrated into current office routines, formally assigned to specific staff members, and supported by internal systems that ensure adequate tracking and follow up of smokers. Ongoing staff training and patient self-management techniques are also needed to ease the introduction of new programs and increase their acceptability to smokers. This is the first study to examine in-depth the organizational changes needed to implement a system for fax referral services in medical settings. Our findings strongly suggest the need for further research examining challenges to the sustainability of tobacco treatment interventions [7,43], as well as funding and resources to support systems changes and fax referrals for primary care practices [39,40,42,44,45].

## Competing interests

The authors declare that they have no competing interests.

## Authors' contributions

DS designed the study. JC and DS conducted the focus groups and interviews. JC completed the literature review for the manuscript. JC and DS coded and analyzed the interview data. JC wrote the initial manuscript. JC and DS provided substantial subsequent contributions. Both authors read and approved the final manuscript.

## Pre-publication history

The pre-publication history for this paper can be accessed here:

http://www.biomedcentral.com/1471-2296/10/81/prepub

## References

[B1] Fiore MC Jaen CR Baker TB Treating Tobacco Use and Dependence: 2008 Update 2008 Department of Health and Human Services. Public Health Service. Rockville, MD

[B2] Orleans CT Woolf SH Rothemich SF Marks JS Isham GJ The top priority: building a better system for tobacco-cessation counseling American Journal of Preventive Medicine 2006 31 1 103 106 10.1016/j.amepre.2006.03.015 16777550

[B3] Mullins S Fagan HB Reed JF Bercaw D Ask and act: Delaware physicians demonstrate the effectiveness of the American Academy of Family Physicians' initiative to promote tobacco cessation counseling Delaware Medical Journal 2009 81 4 155 160 19552219

[B4] Stange KC Woolf SH Gjeltema K One minute for prevention: the power of leveraging to fulfill the promise of health behavior counseling American Journal of Preventive Medicine 2002 22 4 320 323 10.1016/S0749-3797(02)00413-0 11988386

[B5] Revell CC Schroeder SA Simplicity matters: using system-level changes to encourage clinician intervention in helping tobacco users quit Nicotine & Tobacco Research 2005 7 Suppl 1 S67 69 10.1080/14622200500078162 16036272

[B6] Schroeder SA What to do with a patient who smokes JAMA 2005 294 4 482 487 10.1001/jama.294.4.482 16046655

[B7] Curry SJ Orleans CT Keller P Fiore M Promoting smoking cessation in the healthcare environment: 10 years later American Journal of Preventive Medicine 2006 31 3 269 272 10.1016/j.amepre.2006.05.003 16905041

[B8] Draft results from the 2008 North American Quitline Consortium annual survey http://www.naquitline.org/resource/resmgr/survey_2008/2008surveynotespres.pdf

[B9] Marcy TW Solomon LJ Dana GS Secker-Walker R Skelly JM A smoking cessation telephone resource: feasibility and preliminary evidence on the effect on health care provider adherence to smoking cessation guidelines Tobacco Control 2002 11 1 84 10.1136/tc.11.1.84 11891379 PMC1747635

[B10] Perry RJ Keller PA Fraser D Fiore MC Fax to quit: a model for delivery of tobacco cessation services to Wisconsin residents Wisconsin Medical Journal 2005 104 4 37 40 44 16117232

[B11] Bentz CJ Bayley KB Bonin KE Fleming L Hollis JF McAfee T The feasibility of connecting physician offices to a state-level tobacco quit line American Journal of Preventive Medicine 2006 30 1 31 37 10.1016/j.amepre.2005.08.043 16414421

[B12] Anderson CM Zhu SH Tobacco quitlines: looking back and looking ahead Tobacco Control 2007 16 Suppl 1 i81 86 10.1136/tc.2007.020701 18048638 PMC2598521

[B13] Ebbert JO Carr AB Patten CA Morris RA Schroeder DR Tobacco use quitline enrollment through dental practices: a pilot study Journal of the American Dental Association 2007 138 5 595 601 17473036 10.14219/jada.archive.2007.0229

[B14] Mahabee-Gittens EM Gordon JS Krugh ME Henry B Leonard AC A smoking cessation intervention plus proactive quitline referral in the pediatric emergency department: a pilot study Nicotine & Tobacco Research 2008 10 12 1745 1751 10.1080/14622200802443494 PMC4161202 19023825

[B15] Bernstein SL Jearld S Prasad D Bax P Bauer U Rapid implementation of a smokers' quitline fax referral service in an urban area Journal of Health Care for the Poor and Underserved 2009 20 1 55 63 10.1353/hpu.0.0112 19202246

[B16] Willett JG Hood NE Burns EK Swetlick JL Wilson SM Lang DA Levinson AH Clinical faxed referrals to a tobacco quitline: reach, enrollment, and participant characteristics American Journal of Preventive Medicine 2009 36 4 337 340 10.1016/j.amepre.2008.12.004 19201150

[B17] Wagner EH Austin BT Von Korff M Organizing care for patients with chronic illness Milbank Quarterly 1996 74 4 511 544 10.2307/3350391 8941260

[B18] Wagner EH Chronic disease management: what will it take to improve care for chronic illness? Effective Clinical Practice 1998 1 1 2 4 10345255

[B19] Wagner EH Austin BT Davis C Hindmarsh M Schaefer J Bonomi A Improving chronic illness care: translating evidence into action Health Affairs (Millwood) 2001 20 6 64 78 10.1377/hlthaff.20.6.64 11816692

[B20] Bodenheimer T Wagner EH Grumbach K Improving primary care for patients with chronic illness: the chronic care model, Part 2 JAMA 2002 288 15 1909 1914 10.1001/jama.288.15.1909 12377092

[B21] Bodenheimer T Wagner EH Grumbach K Improving primary care for patients with chronic illness JAMA 2002 288 14 1775 1779 10.1001/jama.288.14.1775 12365965

[B22] Glasgow RE Orleans CT Wagner EH Does the chronic care model serve also as a template for improving prevention? Milbank Quarterly 2001 79 4 579 612 iv-v 10.1111/1468-0009.00222 11789118 PMC2751207

[B23] Hung DY Rundall TG Tallia AF Cohen DJ Halpin HA Crabtree BF Rethinking prevention in primary care: applying the chronic care model to address health risk behaviors Milbank Quarterly 2007 85 1 69 91 10.1111/j.1468-0009.2007.00477.x 17319807 PMC2690311

[B24] Carlini BH Schauer G Zbikowski S Thompson J Using the chronic care model to address tobacco in health care delivery organizations: a pilot experience in Washington State Health Promotion Practice 2009 in press 19129434 10.1177/1524839908328999

[B25] Hung DY Shelley DR Multilevel analysis of the chronic care model and 5A services for treating tobacco use in urban primary care clinics Health Services Research 2009 44 1 103 127 10.1111/j.1475-6773.2008.00896.x 18783454 PMC2669639

[B26] Addison RB Crabtree BF, Miller WL A grounded hermeneutic editing approach Doing Qualitative Research 1999 2 Thousand Oaks, Calif.: Sage Publications 145 161

[B27] Sherman SE Takahashi N Kalra P Gifford E Finney JW Canfield J Kelly JF Joseph GJ Kuschner W Care coordination to increase referrals to smoking cessation telephone counseling: a demonstration project American Journal of Managed Care 2008 14 3 141 148 18333706

[B28] Bradley EH Webster TR Baker D Schlesinger M Inouye SK Barth MC Lapane KL Lipson D Stone R Koren MJ Translating research into practice: speeding the adoption of innovative health care programs 2004 New York: Commonwealth Fund 1 12 15270051

[B29] Cohen DJ Tallia AF Crabtree BF Young DM Implementing health behavior change in primary care: lessons from prescription for health Annals of Family Medicine 2005 3 Suppl 2 S12 19 10.1370/afm.334 16049075 PMC1466976

[B30] Green LA Cifuentes M Glasgow RE Stange KC Redesigning primary care practice to incorporate health behavior change: prescription for health round-2 results American Journal of Preventive Medicine 2008 35 5 Suppl S347 349 10.1016/j.amepre.2008.08.013 18929980

[B31] Blumenthal DS Barriers to the provision of smoking cessation services reported by clinicians in underserved communities Journal of the American Board of Family Medicine 2007 20 3 272 279 10.3122/jabfm.2007.03.060115 17478660

[B32] Holtrop JS Malouin R Weismantel D Wadland WC Clinician perceptions of factors influencing referrals to a smoking cessation program BMC Family Practice 2008 9 18 10.1186/1471-2296-9-18 18373854 PMC2323376

[B33] Etz RS Cohen DJ Woolf SH Holtrop JS Donahue KE Isaacson NF Stange KC Ferrer RL Olson AL Bridging primary care practices and communities to promote healthy behaviors American Journal of Preventive Medicine 2008 35 5 Suppl S390 397 10.1016/j.amepre.2008.08.008 18929986

[B34] Kilbourne AM Schulberg HC Post EP Rollman BL Belnap BH Pincus HA Translating evidence-based depression management services to community-based primary care practices Milbank Quarterly 2004 82 4 631 659 10.1111/j.0887-378X.2004.00326.x 15595945 PMC2690181

[B35] Litt J How to provide effective smoking cessation advice in less than a minute without offending the patient Australian Family Physician 2002 31 12 1087 1094 12516509

[B36] Keller PA Fiore MC Curry SJ Orleans CT Systems change to improve health and health care: lessons from addressing tobacco in managed care Nicotine & Tobacco Research 2005 7 Suppl 1 S5 8 10.1080/14622200500077966 16036270

[B37] Aspy CB Mold JW Thompson DM Blondell RD Landers PS Reilly KE Wright-Eakers L Integrating screening and interventions for unhealthy behaviors into primary care practices American Journal of Preventive Medicine 2008 35 5 Suppl S373 380 10.1016/j.amepre.2008.08.015 18929984

[B38] Cohen DJ Crabtree BF Etz RS Balasubramanian BA Donahue KE Leviton LC Clark EC Isaacson NF Stange KC Green LW Fidelity versus flexibility: translating evidence-based research into practice American Journal of Preventive Medicine 2008 35 5 Suppl S381 389 10.1016/j.amepre.2008.08.005 18929985

[B39] Tobacco cessation quitlines - a good investment to save lives, decrease direct medical costs and increase productivity http://www.naquitline.org/resource/resmgr/docs/naqc_issuepaper_tobaccocessa.pdf

[B40] The role of reimbursement and third party financial support in sustaining quitlines http://www.naquitline.org/resource/resmgr/docs/naqc_issuepaper_thirdpartyre.pdf

[B41] Miller N Frieden TR Liu SY Matte TD Mostashari F Deitcher DR Cummings KM Chang C Bauer U Bassett MT Effectiveness of a large-scale distribution programme of free nicotine patches: a prospective evaluation Lancet 2005 365 9474 1849 1854 10.1016/S0140-6736(05)66615-9 15924980

[B42] Orleans CT Increasing the demand for and use of effective smoking-cessation treatments reaping the full health benefits of tobacco-control science and policy gains--in our lifetime American Journal of Preventive Medicine 2007 33 6 Suppl S340 348 10.1016/j.amepre.2007.09.003 18021909

[B43] Curry SJ Keller PA Orleans CT Fiore MC The role of health care systems in increased tobacco cessation Annual Review of Public Health 2008 29 411 428 10.1146/annurev.publhealth.29.020907.090934 18173387

[B44] An LC Bluhm JH Foldes SS Alesci NL Klatt CM Center BA Nersesian WS Larson ME Ahluwalia JS Manley MW A randomized trial of a pay-for-performance program targeting clinician referral to a state tobacco quitline Archives of Internal Medicine 2008 168 18 1993 1999 10.1001/archinte.168.18.1993 18852400

[B45] Dodoo MS Lesser LI Phillips RL Jr Bazemore AW Petterson SM Xierali I Changing patient health-risk behavior requires new investment in primary care American Family Physician 2008 78 8 924 18953968

